# Stimulation of Monocytes by Placental Microparticles Involves Toll-Like Receptors and Nuclear Factor Kappa-Light-Chain-Enhancer of Activated B Cells

**DOI:** 10.3389/fimmu.2014.00173

**Published:** 2014-04-15

**Authors:** Marianne Simone Joerger-Messerli, Irene Mathilde Hoesli, Corinne Rusterholz, Olav Lapaire

**Affiliations:** ^1^Laboratory for Prenatal Medicine, Department of Biomedicine, University Hospital Basel, Basel, Switzerland; ^2^Department of Obstetrics and Gynecology, University Hospital Basel, Basel, Switzerland

**Keywords:** human pregnancy, inflammation, STBM, monocytes, NF-κB, TLR

## Abstract

Human pregnancy is accompanied by a mild systemic inflammatory response, which includes the activation of monocytes circulating in maternal blood. This response is exaggerated in preeclampsia, a placental-dependent disorder specific to human pregnancies. We and others showed that placental syncytiotrophoblast membrane microparticles (STBM) generated *in vitro* from normal placentas stimulated peripheral blood monocytes, which suggest a contribution of STBM to the systemic maternal inflammation. Here, we analyzed the inflammatory potential of STBM prepared from preeclamptic placentas on primary monocytes and investigated the mode of action *in vitro*. STBM generated *in vitro* by placental villous explants of normal or preeclamptic placentas were co-incubated with human peripheral blood monocytes. In some cases, inhibitors of specific cellular functions or signaling pathways were used. The analysis of the monocytic response was performed by flow cytometry, enzyme-linked immunoassays, real-time PCR, and fluorescence microscopy. STBM derived from preeclamptic placentas up-regulated the cell surface expression of CD54, and stimulated the secretion of the pro-inflammatory interleukin (IL)-6 and IL-8 in a similar, dose-dependent manner as did STBM prepared from normal placentas. STBM bound to the cell surface of monocytes, but phagocytosis was not necessary for activation. STBM-induced cytokine secretion was impaired in the presence of inhibitors of toll-like receptor (TLR) signaling or when nuclear factor kappa-light-chain-enhancer of activated B cells (NF-κB) activation was blocked. Our results suggest that the inflammatory reaction in monocytes may be initiated by the interaction of STBM with TLRs, which in turn signal through NF-κB to mediate the transcription of genes coding for pro-inflammatory factors.

## Introduction

Preeclampsia is a multi-symptom disorder of the second half of pregnancy, which affects 2–7% of pregnant women worldwide (1). Despite extensive research, the etiology of this pathologic pregnancy condition remains unclear. The current research suggests that, in patients destined to develop preeclampsia, pregnancy is associated with an increased maternal inflammatory reaction, which will lead to placental stress and ultimately result in the mother’s systemic endothelial dysfunction and the large array of life-threatening symptoms, which characterize the disorder (2–4). Interestingly, normal pregnancy also induces a physiologic systemic inflammatory response toward term, however in a much milder form as found in preeclampsia (5).

On the other hand, it is well-acknowledged that the placenta plays a crucial role in the development of the systemic maternal symptoms of preeclampsia. Over the years, evidence for abnormal development, function, and tissue turnover of the placenta has accumulated (6– 8). We and others have suggested that placenta-derived syncytiotrophoblast membrane microparticles (STBM), which are shed into maternal blood in higher amounts in preeclampsia as compared to normotensive pregnancies (9), may play an active role in stimulating the mother’s inflammatory response (10– 12). *In vitro*, STBM interfere with human umbilical vein endothelial cell proliferation and survival or with the relaxation of artificially pre-constricted small subcutaneous fat arteries (13– 16). In addition, STBM induce a strong pro-inflammatory response in donor-derived human peripheral polymorphonuclear leukocytes (17) and in human peripheral blood monocytes (18, 19). In monocytes, the production of the pro-inflammatory cytokines tumor necrosis factor (TNF) α, interleukin (IL)-12, IL-6 and of the chemokine IL-8 is increased, whereas the cells also adopt a cell surface expression pattern with up-regulation of the adhesion molecule CD54, which is very reminiscent of the pattern exhibited by peripheral blood monocytes freshly harvested from pregnant women (20). Compared to non-pregnant controls, circulating monocytes from healthy pregnant women display an inflammatory phenotype, which is hallmarked by an enhanced phagocytic activity, elevated basal production of reactive oxygen species (21), and increased production of pro-inflammatory mediators (22). In preeclampsia, peripheral monocytes are more extensively activated when compared to their counterparts in normal pregnancy, with a further increase in the production of IL-1β, IL-6, and IL-8 (23). These latter features concur with our previous results indicating that STBM generated from normal placentas had the potential to stimulate monocytes in a dose-dependent manner (19). Therefore, we suggested that the progressive monocytic activation in the maternal peripheral blood during pregnancy might be due to the steady increase in the amount of placental microparticles as gestational age advances. We also proposed that the excessive monocytic activation in preeclampsia might be correlated with the elevated circulatory STBM concentrations existing in this pregnancy condition. However, evidence that microparticles shed from preeclamptic placentas similarly stimulate monocytes is still scarce. Interestingly, plasma-derived microparticles from preeclamptic women activate endothelial cells *in vitro*, in the presence of monocytes, to a higher extent than microparticles isolated from normotensive women (24). This study did however not identify the specific subgroup of microparticles, which affected the co-cultures of endothelial cells with monocytes and did not detail the monocytic contribution to endothelial cell activation. Therefore, in the present study, we prepared STBM *in vitro* by explant cultures of preeclamptic placentas and investigated their effect on human peripheral blood monocytes.

## Materials and Methods

### *In vitro* generation of STBM

This study was approved by the local ethical committee (Cantonal Institutional Review Board of Basel, Switzerland). In all cases, written informed consent was received. Human term placentas from uncomplicated pregnancies and placentas from cases with preeclampsia were collected in the Department of Obstetrics and Gynecology, University Hospital of Basel, within 1 h following elective or secondary cesarean section. Explants from villous tissue were set in culture in a controlled atmosphere (37°C, 20% oxygen/5% carbon dioxide) as described previously (19). STBM were isolated from the culture supernatants by a three-step centrifugation procedure at 4°C, namely 1000 × *g* for 10 min, 10,000 × *g* for 10 min, and 60,000 × *g* for 90 min. The microparticle-containing pellet was washed with PBS, re-suspended in PBS/5% sucrose and stored at −20°C until use. The protein content of the STBM was quantified with the Advanced Protein Assay Reagent (Cytoskeleton Inc., Denver, CO, USA) and STBM were standardized for protein concentrations as indicated in the figure legends.

### Isolation of human monocytes

Forty milliliters of venous blood from healthy male donors, which were collected in EDTA-containing tubes, were centrifuged on Histopaque (Sigma, Saint Louis, MO, USA) density gradient according to the manufacturer’s instructions. Peripheral blood mononuclear cells (PBMCs) were washed twice with PBS supplemented with 2 mM EDTA and the residual erythrocytes were lysed with the red blood cell lysis solution (Qiagen, Valencia, CA, USA). Monocytes were isolated through negative selection by means of the Human Monocyte Isolation Kit II and a magnetic cell separation system (MACS), according to the manufacturer’s instructions (Miltenyi Biotec Inc., Auburn, CA, USA).

### Co-culture of monocytes and STBM

Monocytes were cultured in a final concentration of 5 × 10^5^ cells/ml in RPMI-1640 (Gibco, Grand Island, NY, USA) supplemented with 10% FCS (Amimed, Allschwil, Switzerland), 4 mM glutamine (Gibco), and 100 U/ml penicillin/streptomycin (Gibco). Cells were either left untreated or co-incubated with different amounts of STBM as mentioned in the figure legends, or stimulated with lipopolysaccharide (LPS) from Gram-negative bacteria (Sigma) as positive control. In some experiments, monocytes were pre-treated for 15 min with the phagocytosis inhibitor cytochalasin B (Sigma), or the NF-κB inhibitors 6-amino-4-(4-phenoxyphenylethylamino) quinazoline (Calbiochem, San Diego, CA, USA) and perillyl alcohol (PA) (Sigma), or for 24 h with a peptide inhibiting myeloid differentiation response gene (MyD) 88 homodimerization, before addition of STBM. Co-cultures were incubated during 4 h (RNA analysis), 12 h (cytokine analysis after the inhibition of MyD88 homodimerization), or 16 h (flow cytometry and cytokine analysis) at 37°C in 20% oxygen/5% carbon dioxide. At the end of the culture, the cells and the culture supernatants were separately harvested for further analysis. Cell viability was confirmed with the Cell Proliferation Reagent WST-1 (Roche Diagnostics GmbH; Mannheim, Germany).

### Flow cytometry

Monocytes were first incubated with 200 μg/ml of purified human IgG (Sigma) in PBS supplemented with 2 mM EDTA and 1% FCS for 5 min at 4°C to prevent unspecific binding through Fc receptors. Incubations with specific antibodies were carried out for 15 min at 4°C with ready-to-use concentrations of FITC-conjugated antibodies against CD14 (BD Pharmingen, San Jose, CA, USA) and PE-conjugated antibodies against CD54 (BD Pharmingen) or APC-conjugated antibody against CD11a (BD Pharmingen). Labeled cells were washed with PBS and re-suspended in PBS supplemented with 2 mM EDTA and 0.5% BSA. The data was acquired on a Dako Cyan flow cytometer (Beckman Coulter, Fullerton, CA, USA) and analyzed with the Summit software.

### Enzyme-linked immunosorbent assay

The secreted levels of IL-8 and IL-6 were measured in duplicates using the respective DuoSet^®^ enzyme-linked immunosorbent assay (ELISA) development kits (R&D Systems Inc., Minneapolis, MN, USA), according to the manufacturer’s protocol. Plates were read at 450 nm with a wavelength correction set at 562 nm, in the Spectramax 250 microplate reader (Molecular Devices, Sunnyvale, CA, USA) and analyzed with Softmax Pro software (Molecular Devices).

### Fluorescent microscopy

Purified monocytes were incubated in 1 μM carboxyfluorescein succinimidyl ester (CFSE) (kindly provided by Prof. G. Spagnoli, Department of Biomedicine, University Hospital of Basel) in the dark at 37°C for 10 min. The staining reaction was stopped with 2 ml of complete RPMI-1640 culture medium. Cells were washed three times with PBS and re-suspended in complete RPMI-1640 culture medium. STBM were diluted 1:10 in diluent C and incubated with 5 μM PKH26, using the PKH26 Red Fluorescent Cell Linker kit (Sigma). After 5 min of incubation at RT, the staining was stopped with 2 ml FCS (Amimed, Allschwil, Switzerland). STBM were washed with PBS and re-suspended in PBS/5% sucrose. The CFSE-labeled monocytes (5 × 10^5^ cells/ml) were co-cultured with 100 μg/ml PKH26-labeled STBM for 16 h at 37°C. Cells were then harvested, washed with PBS, and transferred by cyto-centrifugation onto a glass slide (Shandon, Frankfurt, Germany). Slides were dried at RT in the dark, fixed with 4% formaldehyde (Sigma) for 30 s, counterstained with 0.01% DAPI/Glycerol (Fluka Chemie GmbH, Buchs, Switzerland), and immediately examined with an Axioplan 2 imaging fluorescent microscope using the appropriate filters (Carl Zeiss, Zürich, Switzerland).

### RNA extraction and reverse transcription

Monocytes were washed with ice-cold PBS and lysed in 500 μl Trizol reagent (Gibco). After centrifugation at 12,000 × *g* at 4°C, the aqueous phase was collected and 0.5 volume of ice-cold EtOH was added. RNA was then isolated using the RNAeasy Mini kit (Qiagen, Valencia, CA, USA) according to the manufacturer’s protocol. The concentration of RNA was determined by spectrophotometry (NanoDrop Technologies, Wilmington, DE, USA). One hundred sixty seven nanograms of RNA were reverse-transcribed using 500 ng random primers included in the Reverse Transcription System (Promega Corporation, Madison, WI, USA). The reaction was performed on a TRIO-Thermoblock (Biometra, Goettingen, Germany) under the following conditions: 10 min at 37°C, 60 min at 45°C, 5 min at 95°C, and 15 min at 4°C.

### Real-time PCR

The human NF-κB signaling pathway RT^2^ profiler PCR array is a commercially available real-time PCR based assay (SABiosciences, Frederick, MD, USA), which profiles the expression of 84 key genes involved in the NF-κB signaling transduction. The plates were received pre-coated with forward and reverse primers of the respective genes. For one 96-well plate, 102 μl of cDNA was mixed with 1275 μl of 2× SuperArray RT^2^ qPCR master mix and filled up with H_2_O to the final volume of 2550 μl. Twenty-five microliters of the experimental cocktail were pipetted into each well of the PCR array. The PCR was run with the following PCR cycling program on ABI PRISM^®^ 7000 Sequence Detection System (Applied Biosystems Inc., Forster City, CA, USA): 10 min at 95°C, followed by 40 cycles of 15 s at 95°C and 1 min at 60°C. The data were analyzed with the delta–delta *C*_t_ method using the online software provided on the company’s webpage and expressed as fold change $\left(2^{-\Delta \Delta C_{t}}\right)$ relative to the untreated cells.

### Statistical analysis

To calculate the significance of differences between the experimental groups, the Mann–Whitney test was performed using SPSS 15.0 (Statistical Package for the Social Sciences; Chicago, IL, USA). *p* < 0.05 was considered to be statistically significant.

## Results

### STBM generated by explant cultures of preeclamptic placentas activate human peripheral blood monocytes in a similar way as STBM derived from normal placentas (STBM-NP)

Since only a minor fraction of all microparticles circulating in the blood of healthy pregnant women are shed from the placenta (25), STBM are usually generated *in vitro*. Recently, we established short-term explant cultures of villous tissue to produce STBM-NP (14). Here, this method was applied on placentas, which were collected from women with preeclampsia.

**Figure 1:**
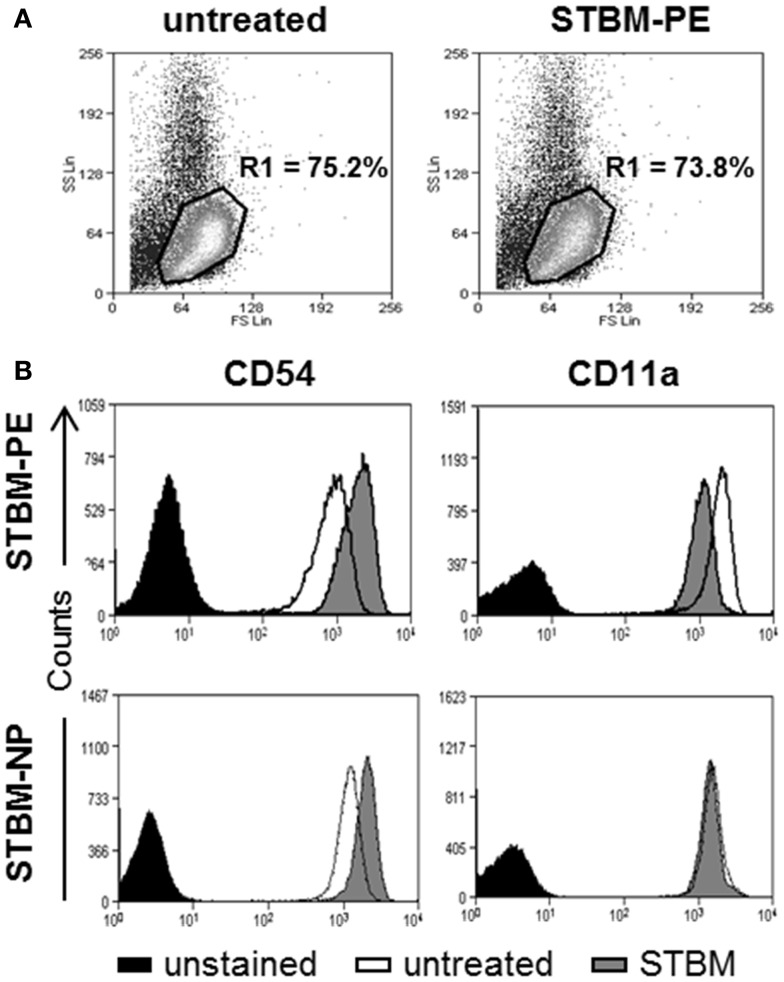
**STBM from normal and preeclamptic placentas alter the cell surface expression of CD54 and CD11a on monocytes**. Primary human monocytes were left untreated or were co-cultured with 300 μg/ml STBM prepared from normal (STBM-NP) or preeclamptic (STBM-PE) placentas for 16 h. The cell surface expression of CD54 and CD11a was evaluated on CD14-positive cells using flow cytometry. **(A)** Representative forward/side scatter dot plots of untreated or STBM-treated cells. **(B)** Representative histograms of CD54 or CD11a expression on untreated or STBM-treated monocytes. The co-culture experiments were performed two times with five different STBM-NP preparations and three independent STBM-PE preparations.

To investigate if the microparticles generated from the preeclamptic placentas (STBM-PE) altered the expression profile of the inflammatory markers CD54 and CD11a on primary human monocytes, the cells were incubated with STBM-PE for 16 h and cell surface expression was monitored by flow cytometry. More than 95% of the cells expressed the monocyte-specific marker CD14 and its expression remained unchanged with incubation with the microparticles (Figure S1 in Supplementary Material). On the contrary, the expression levels of CD54 were significantly increased [mean increase of median fluorescence intensity (MFI) ± SEM: 641.2 ± 122.5, *p* < 0.01] and the levels of CD11a were significantly decreased (mean decrease of MFI ± SEM: 583.4 ± 77.2, *p* < 0.01) following co-culture with STBM-PE (Figure 1). This was very comparable to the changes triggered by STBM-NP, except for CD11a, which was only modestly affected by the latter (Figure 1B, lower panels).

**Figure 2:**
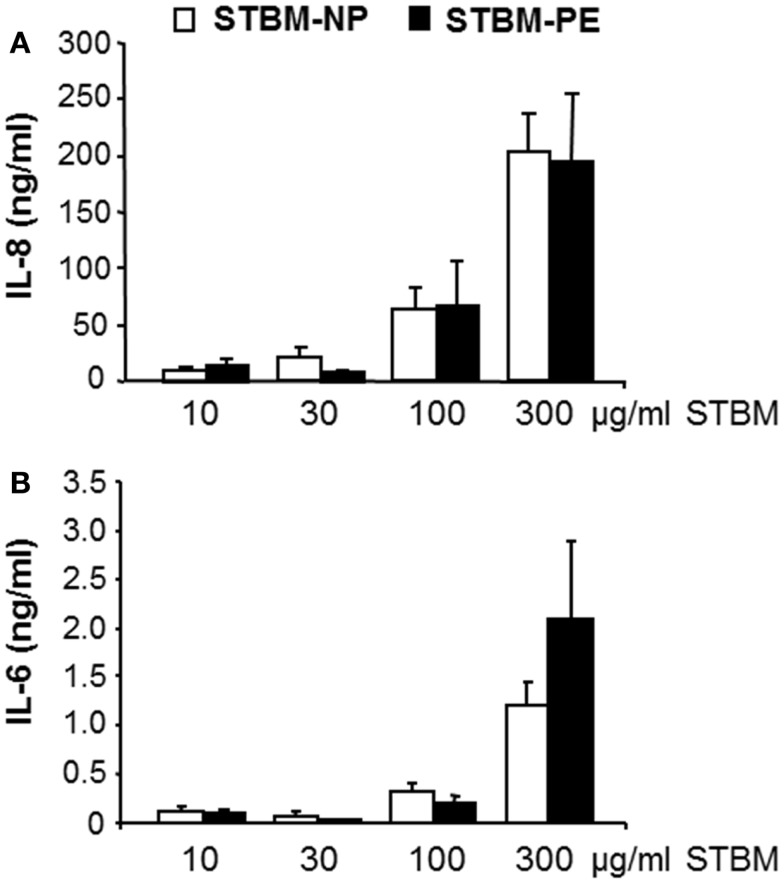
**STBM produced from normal and preeclamptic placentas induce a similar dose-dependent pro-inflammatory response in monocytes**. Primary monocytes were incubated with increasing concentrations of STBM prepared from normal (STBM-NP) or preeclamptic (STBM-PE) placentas for 16 h. The cellular secretion of IL-8 **(A)** and IL-6 **(B)** was measured by conventional ELISA. Results are illustrated as mean ± SEM of two independent monocyte co-culture experiments with five STBM-NP preparations and three STBM-PE preparations. There is no statistical difference between the responses generated by the two different STBM populations.

In our previous study, we showed that STBM-NP stimulated primary monocytes to secrete soluble mediators of inflammation (19). Therefore, the effect of STBM-PE on the monocytic production of the pro-inflammatory cytokine IL-6 and the chemokine IL-8 was investigated. This analysis showed that STBM-PE stimulated the secretion of IL-6 and IL-8 in a dose-dependent manner, as did STBM-NP. Furthermore, both microparticle populations induced similar concentrations of cytokines at identical doses (Figure 2). However, a higher sample number would be required to test for the absence of a difference.

### Monocytic activation does not require phagocytosis of the STBM

**Figure 3:**
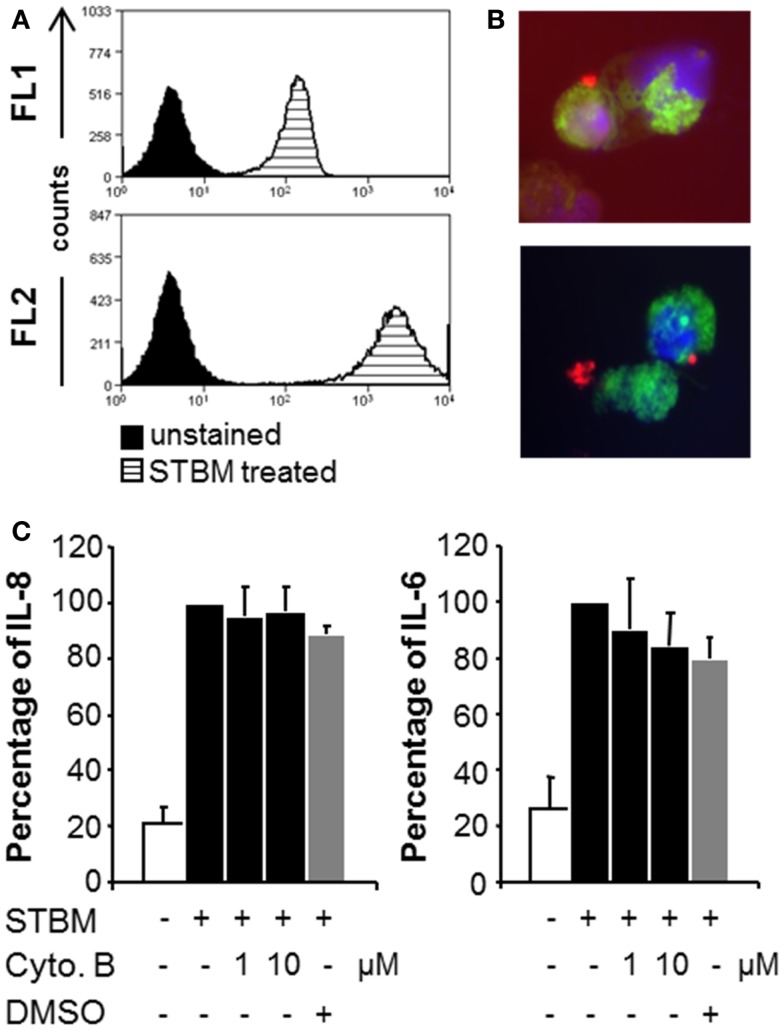
**STBM interact with monocytes, but phagocytosis is not required for their stimulatory properties**. PKH-26-labeled STBM-NP (300 μg/ml) were incubated with monocytes for 16 h and analyzed by flow cytometry or fluorescence microscopy. **(A)** Representative histograms of CD14-positive monocytes read in the FL1 (CD14) and FL2 (PKH-26) channels. **(B)** Fluorescence microscopy of CSFE-stained monocytes (green) incubated with PKH-labeled STBM (red). DAPI (blue) was used as a nuclear counterstain. **(C)** Monocytes were pre-treated with various concentrations of cytochalasin B in DMSO or with DMSO alone, prior to the addition of 300 μg/ml STBM. The levels of IL-8 and IL-6 secreted by the cells after 16 h of incubation are expressed as a percentage of the amounts produced by cells stimulated with STBM in the absence of the inhibitor, which was set at 100%. Data represent mean ± SEM of three different co-cultures with STBM-NP prepared from three placentas. There was no statistical difference between cytokine secretion in the absence or in the presence of cytochalasin B.

As both populations of STBM triggered similar inflammatory responses in primary monocytes, the molecular mechanisms of cell activation were addressed using the more available STBM-NP. To assess whether placental microparticles could interact with human monocytes, they were labeled with the red fluorescent lipophilic dye PKH26 prior incubation with the cells. Flow cytometric analysis revealed that all monocytes became highly positive for PKH26, which indicates that STBM directly interact with the cells (Figure 3A). However, this analysis did not permit to localize precisely the microparticles. Therefore, the experiment was repeated with monocytes pre-treated with the green fluorescent intracellular dye CFSE and PKH26-labeled STBM-NP and analyzed by fluorescence microscopy. STBM were regularly found on the rim of the cells, suggesting that they bind to but are not internalized by the cells (Figure 3B). In some cases, clamps of STBM formed and attached on the cell surface (Figure 3B, lower panel).

To verify that internalization of the placental microparticles was not required to activate the cells, the monocytes were pre-treated with cytochalasin B, a cell-permeable mycotoxin that blocks phagocytosis, prior addition of the microparticles. Neither cytochalasin B nor the drug-carrier DMSO interfered with the STBM-induced secretion of IL-8 and IL-6 (Figure 3C). Cell viability after culture was confirmed (Figure S2 in Supplementary Material).

### Toll-like receptors are involved in the STBM-induced activation of monocytes

**Figure 4:**
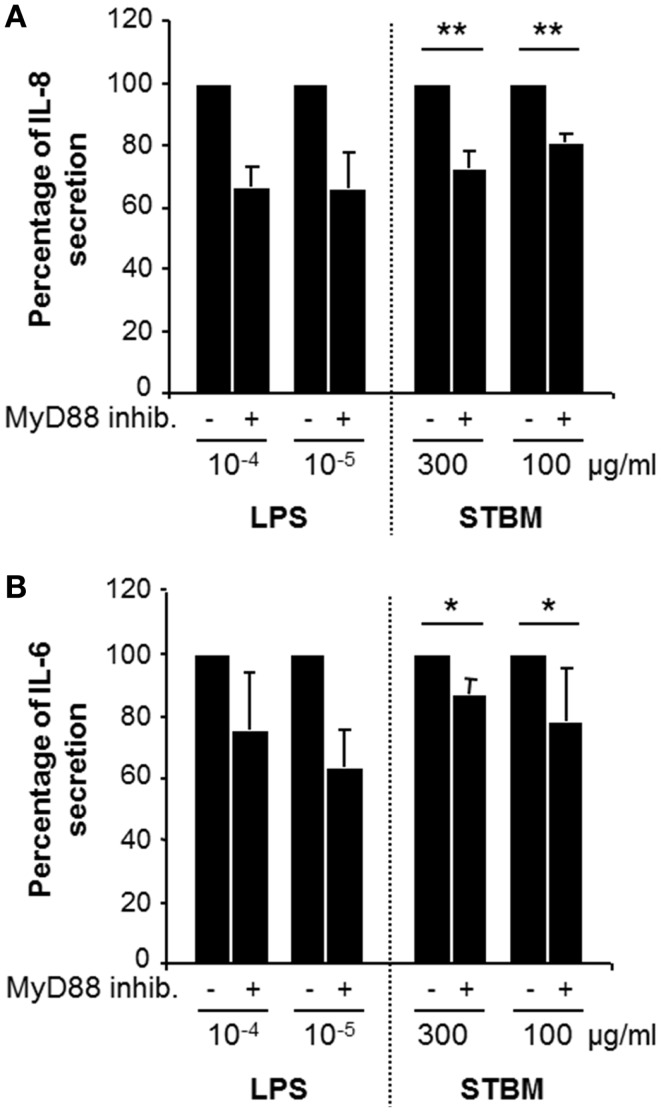
**Inhibition of MyD88 homodimerization reduces STBM-induced pro-inflammatory response**. Primary monocytes were pre-treated with 1 μM MyD88 inhibitor peptide for 24 h prior incubation with the indicated concentrations of LPS or STBM-NP during 12 h. The concentrations of IL-8 **(A)** and IL-6 **(B)** secreted by the cells were quantified by ELISA and are presented as a percentage of the amounts secreted in absence of pre-treatment with the inhibitor. Data represent mean ± SEM of three monocyte co-culture experiments with STBM-NP prepared from four placentas. *p < 0.05; **p < 0.01. LPS was used as a positive control.

As STBM interact with the cell surface of monocytes, we checked whether cell membrane receptors of the toll-like family (TLR) may be involved. Accordingly, monocytes were pre-treated with a peptide inhibiting MyD88 homodimerization, a broad TLR blocking agent, prior incubation with LPS or STBM-NP. LPS transmits intracellular activation signals through TLR4. Pre-treatment of monocytes with MyD88 inhibitory peptide partially impaired LPS-induced secretion of IL-6 and IL-8 (Figure 4). In a similar way, incubation of monocytes with STBM-NP in the presence of the MyD88 inhibitory peptide lead to significantly reduced secretion of both pro-inflammatory mediators compared to cells cultured with STBM-NP alone (Figure 4). Thus, placental microparticles appear to activate monocytes, at least in part, via one or several members of the TLR family.

### STBM activate monocytes in an NF-κB-dependent manner

**Figure 5:**
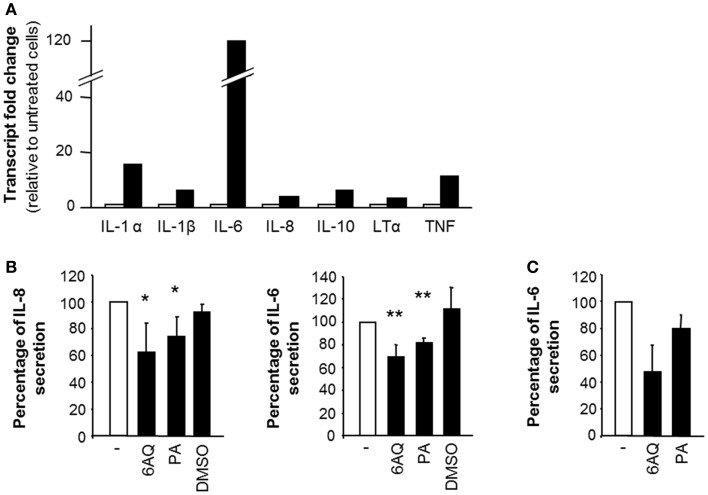
**The effect of STBM on monocytes is partly mediated by NF-κB**. **(A)** Monocytes were incubated during 4 h without or with 300 μg/ml STBM-NP prepared from three different placentas. Cellular RNA was extracted, reverse-transcribed, and the pooled cDNA was examined for the expression of several genes. Transcript fold change was calculated with the $2^{-\Delta \Delta C_{t}}$. **(B,C)** Monocytes were pre-incubated with either 10 μM 6AQ, or 10 μM PA or DMSO alone prior to addition of **(B)** 300 μg/ml STBM-NP or **(C)** 100 pg/ml LPS. After 16 h of incubation, cytokine secretion was measured by ELISA and reported as a percentage of secretion relative to that of monocytes stimulated by STBM-NP in the absence of any drug. Data are presented as mean ± SEM of three independent co-culture experiments with four STBM-NP preparations. *p < 0.05; **p < 0.01. LPS was used as a positive control.

In order to establish whether the secretion of IL-6 and IL-8 in response to STBM stimulation was due to *de novo* gene transcription, we analyzed mRNA levels by relative real-time PCR. IL-6 and IL-8 mRNA levels were increased 120-fold and 4-fold, respectively, upon STBM-treatment compared to untreated monocytes (Figure 5A). The transcription of several other mediators involved in the inflammatory response, such as IL-1α, IL-1β, IL-10, lymphotoxin (LT)-α, and the TNF, was also induced (Figure 5A). Besides stimulating the transcription of several targets, STBM-NP also down-regulated the transcription of molecules involved in apoptosis, like caspase-8 $\left(2^{-\Delta \Delta C_{t}}=0.32\right)$, CD27 $\left(2^{-\Delta \Delta C_{t}}=0.43\right)$, and Fas-associated protein with death domain $\left(\text{FADD};\;2^{-\Delta \Delta C_{t}}=0.5\right)$ (data not shown).

The gene transcription of these pro-inflammatory mediators is controlled in large part by nuclear factor kappa-light-chain-enhancer of activated B cells (NF-κB) (26). Hence, the potential participation of NF-κB in the activated phenotype of STBM-treated monocytes was confirmed using 6AQ and PA, two distinct inhibitors of NF-κB activation. Both inhibitors significantly decreased IL-6 and IL-8 secretion induced by STBM-NP compared to cells co-cultured with the microparticles in the absence of the inhibitors (Figure 5B). DMSO, which was used as a carrier for 6AQ, had no effect on IL-6 or IL-8 release. In all instances, cell viability following treatment with the drugs was confirmed (Figure S2 in Supplementary Material). As positive control, LPS, which activates NF-κB function, was used to stimulate monocytes. The LPS-induced release of IL-6 was also partially reduced by the same concentrations of either NF-κB inhibitors (Figure 5C).

## Discussion

Syncytiotrophoblast microparticles have been attributed potential roles in the systemic maternal inflammatory response during normal pregnancy and in the enhanced inflammatory reaction in preeclampsia (18, 19). In contrast to syncytial knots and larger placental debris, eliciting a local inflammatory response upon phagocytosis by endometrial endothelial cells (27), and which are trapped by alveolar macrophages in the lungs (28, 29), STBM reach the maternal peripheral circulation, where they may come into contact with maternal endothelial and immune cells. Here, we showed that STBM derived from placentas collected from women with diagnosed preeclampsia or from women with uneventful pregnancy had a comparable effect on monocytes. STBM-PE, like STBM-NP, increased the cell surface levels of CD54 and induced the secretion of pro-inflammatory factors on monocytes. The cellular response to either STBM population was dose-dependent and equally strong at equivalent concentrations of microparticles. Our data would therefore indicate that the exaggerated inflammatory reaction in patients with preeclampsia may be attributed to the elevated levels of circulating placental microparticles rather than to a differential nature of the particles in preeclampsia compared to normal pregnancy. However, it needs to be mentioned that STBM-PE significantly altered the cellular expression of CD11a, whereas STBM-NP had no effect on this adhesion molecule. Hence, this could indicate that the adhesion properties of the monocytes may change differently upon their interaction with STBM in preeclamptic patients compared to normotensive pregnant women. Others have shown that microvesicles produced from preeclamptic placentas *in vitro* had an exacerbated pro-inflammatory effect on PBMCs when compared to normal term microvesicles (30), giving a hint that the placental debris shed by preeclamptic placentas may possibly be qualitatively different from the placental micro-debris circulating in the blood of normotensive women. Alluding to this hypothesis, a recent study demonstrated that microparticles derived from a trophoblast cell line cultured under hypoxia, as a model for the preeclamptic placenta, triggered a more rapid inflammatory response in PBMC than the particulate material derived from the cell line cultured under normal oxygen conditions (31). It was also recently shown that the monocytic fraction of PBMC was at the origin of the production of IL-6 and IL-8 upon stimulation with STBM obtained through placental perfusion (32). Whether this effect was direct or mediated by the other immune cells present in PBMC was however not determined.

Here, we observed that STBM localized at the boundary of monocytes, suggesting that the microparticles interact with these cells via one or several cell surface molecules. These results are in line with a former study, which documented the physiologic binding of placental microparticles onto circulating monocytes in peripheral blood of normal pregnant women and patients with preeclampsia (18). Similarly, STBM collected from placental perfusion were shown to bind and to be internalized by monocytes *in vitro* (32).Furthermore, we provide evidence against the requirement for STBM internalization by showing that the phagocytosis inhibitor cytochalasin B did not affect the secretion of IL-6 and IL-8 in response to the microparticles.

Therefore, the activation of monocytes involves receptors encompassed on the cellular membrane. In this respect, TLRs might be potential candidates, as they mediate inflammatory responses in a number of immune cells following stimulation by a large panel of triggers, including host-derived molecules (33). TLRs transduce activation signals through the adaptor protein MyD88 (34). MyD88 is bound to the intracellular domain of the TLRs and, upon receptor stimulation, it homodimerizes and recruits IL-1 receptor-associated kinase, leading to the activation of the transcription factors NF-κB and JNK. We show here that MyD88 homodimerization inhibitory peptide partially blocks the secretion of IL-6 and IL-8, which strongly suggests that one or several members of the TLR family might be involved in the STBM-induced activation of monocytes. Downstream of TLRs, NF-κB is a well-known master switch in intracellular signaling pathways, which regulates the expression of numerous pro-inflammatory genes required to mount a cellular response in immune cells (26). In monocytes, the mRNA levels of a number of NF-κB responsive genes, including IL-6, IL-8, IL-1, LT-α, and TNF, were up-regulated following STBM treatment. The change in mRNA expression was even higher than 100 fold for IL-6. On the contrary, the levels of IL-8 transcripts were only marginally enhanced despite the large amounts of IL-8 that was secreted by the cells. This observation could point to the presence of an intracellular reservoir of pre-stored IL-8 in monocytes, as it is the case in Weibel–Palade bodies in microvascular endothelial cells (35). What may at first hand appear more intriguing is that STBM also induced the expression of the gene coding for the anti-inflammatory cytokine IL-10. This could be part of a negative feedback mechanism to maintain homeostatic control and terminate the inflammatory reaction (36). We also found that STBM decreased transcript levels of the pro-apoptotic molecules FADD, caspase-8, and CD27, which is in agreement with the role of NF-κB in promoting cell survival (37). Both inhibitors of NF-κB function, 6AQ and PA, independently affected cytokine secretion induced by STBM. 6AQ is a cell-permeable quinazoline compound, which inhibits NF-κB transcriptional activation (38), whereas PA is thought to block the calcium-dependent NF-κB signaling (39). It is known that these inhibitors can induce cellular apoptosis, since NF-κB also functions as a cell survival factor. However, the reduced production of IL-6 and IL-8 in the presence of 6AQ or PA was not due to cell death as the viability of the monocytes at the end of the co-culture with the microparticles was confirmed.

Activation of the NF-κB signaling pathway in PBMCs of preeclamptic women remains controversial. On the one hand, an increased activation of NF-κB in PBMC of preeclamptic patients compared to normal pregnant controls was reported (40). On the other hand, evidence for a suppression of the NF-κB activation pathway in preeclampsia was also provided elsewhere (41). However, this suppression might be attributed to the T-cell subset rather than to the monocytes (42).

In conclusion, the current analysis suggests that even though there may be minor qualitative differences between placental microparticles derived from normal or preeclamptic placentas, both can stimulate primary monocytes to produce pro-inflammatory cytokines. This effect appears to be mediated at least in part through TLR and NF-κB, leading to *de novo* gene transcription. Recent compelling investigations have led to the speculation that the size of the placental particulate debris circulating in the maternal blood may also have its importance in preeclampsia (43, 44). Therefore, further investigation will be required to identify the subgroup of placental microparticles and their components, which transmit pro-inflammatory signals to the maternal immune cells in order to complete our understanding of the inflammatory reactions taking place in both normal pregnancy and preeclampsia.

## Conflict of Interest Statement

The authors declare that the research was conducted in the absence of any commercial or financial relationships that could be construed as a potential conflict of interest.

## Supplementary Material

The Supplementary Material for this article can be found online at http://www.frontiersin.org/Journal/10.3389/fimmu.2014.00173/abstract

## Abbreviations

6AQ, 6-amino-4-(4-phenoxyphenylethylamino)quinazoline; MACS, magnetic cell separation system; MyD88, myeloid differentiation primary response gene 88; NF-κB, nuclear factor kappa-light-chain-enhancer of activated B cells; PA, perillyl alcohol; STBM, syncytiotrophoblast membrane microparticles; STBM-NP, STBM derived from normal placentas; STBM-PE, STBM derived from preeclamptic placentas; TLR, toll-like receptor.
