# Supplementary material for: Stimulation of Monocytes by Placental Microparticles Involves Toll-Like Receptors and Nuclear Factor Kappa-Light-Chain-Enhancer of Activated B Cells
Source: Front Immunol. 2014 Apr 15;5:173. doi: 10.3389/fimmu.2014.00173 (PMC3995043; doi:10.3389/fimmu.2014.00173)
Supplement: Supplementary file 1 [file DataSheet1.DOCX]

***Supplementary Material***

**Stimulation of monocytes by placental microparticles involves Toll-like receptors and nuclear factor kappa-light-chain-enhancer of activated B cells**

**Marianne Joerger-Messerli^1†*^, Irene Hoesli^2^, Corinne Rusterholz^1‡^, Olav Lapaire^1,2^**

^1^Laboratory for Prenatal Medicine, Department of Biomedicine, University Hospital Basel, Basel, Switzerland

^2^Department of Obstetrics and Gynecology, University Hospital Basel, Basel, Switzerland

^†^Present address: Laboratory for Prenatal Medicine, Department of Clinical Research, University of Bern, and Department of Obstetrics and Gynecology, Obstetrics and Feto-Maternal Medicine, University Hospital Bern, Bern, Switzerland

^‡^Present address: Swiss Group for Clinical Cancer Research, SAKK Coordinating Center, Bern, Switzerland

*** Correspondence:** Marianne Joerger-Messerli, Laboratory for Prenatal Medicine, Department of Clinical Research, KKL D/P3 / University Hospital Bern, CH-3010 Bern, Switzerland

marianne.joerger@dkf.unibe.ch

1. **Supplementary Data**

Cell viability assay:

After co-culture, monocytes were washed and resuspended in 100 µl fresh complete medium and put in a 96-well plate. WST-1 reagent (Roche Diagnostics GmbH, Mannheim, Germany) was added (10 µl/well) and incubated 2 h at 37 °C. Optical density (OD) was measured at 450nm, corrected by the reference wavelength of 600nm (Spectramax 250 microplate spectrometer).

1. **Supplementary Figures**

## Suplementary Figures


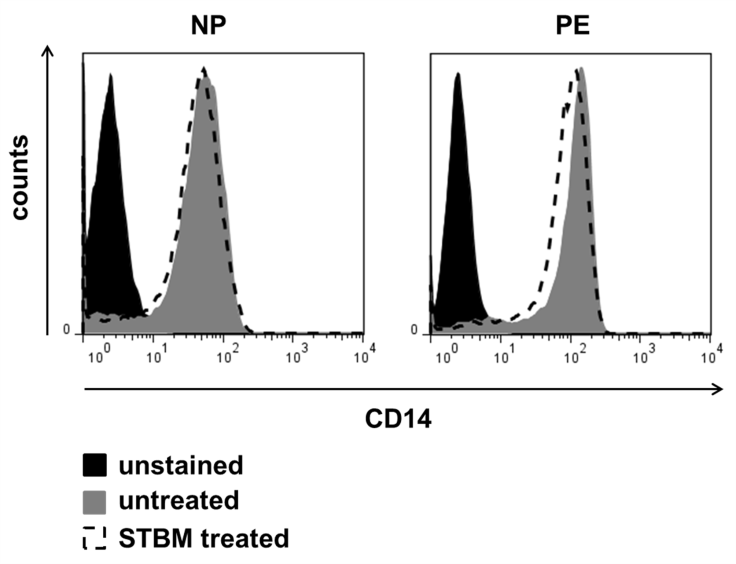


Supplementary Figure 1. STBM treatment do not change CD14 expression on human monocytes. Monocytes were either left untreated or incubated with 300 µg/ml STBM for 16 h, and analyzed for the expression of CD14 by flow cytometry. Data are presented as representative histograms (FlowJo software). The co-culture experiments were done two times with 5 independent STBM-NP (STBM prepared from tormal placentas) and 3 different STBM-PE (STBM generated from preeclamptic placentas) preparations.


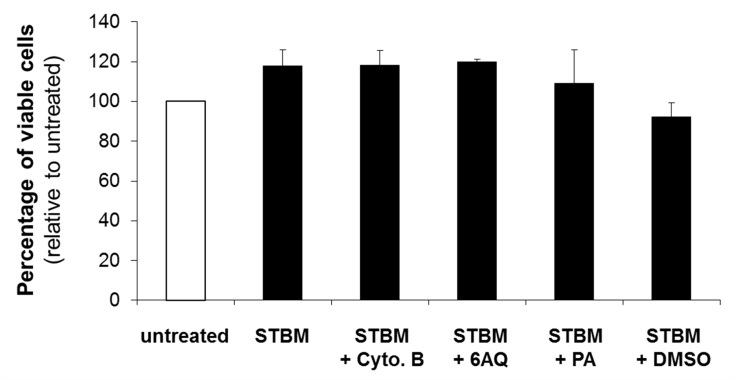


Supplementary Figure 2. Cell viability following treatment with drugs and DMSO was confirmed. After the co-culture of monocytes with 300 µg/ml STBM alone or with 10 µM cytochalasin B, 10 µM 6AQ, 10 µM PA or 0.1% DMSO, cell viability was assessed using WST-1 reagent and expressed as percentage of viable cells relative to untreated cells. The experiments were performed with 3 different STBM preparations. 6AQ = 6-amino-4-(4-phenoxyphenylethylamino)quinazoline; PA = perillyl alcohol.
